# Ti_2_O-Based Saturable Absorbers: From Material Synthesis to Broadband Mode-Locked Fiber Laser Applications

**DOI:** 10.3390/nano16130798

**Published:** 2026-06-27

**Authors:** Guokai Dai, Yuanxiao Qu, Jinjuan Cheng, Chengcheng He, Wei Xu, Luo Yan, Jia Guo

**Affiliations:** 1 School of Mathematics and Physics, University of South China, Hengyang 421001, China; 20242007210438@stu.usc.edu.cn (G.D.); chengjinjuan@usc.edu.cn (J.C.); 2025001089@usc.edu.cn (C.H.); weixu@usc.edu.cn (W.X.); 2 Key Laboratory of Advanced Technologies of Materials, Southwest Jiaotong University, Chengdu 610031, China; qyx2731102023@my.swjtu.edu.cn

**Keywords:** Ti_2_O, saturable absorber, mode-locking, Yb-doped fiber laser, Er-doped fiber laser

## Abstract

Saturable absorbers (SAs) are critical for passive mode-locking in ultrafast fiber lasers. Although many materials have been studied as SAs, new candidates with broadband and stable performance are still needed. In this work, we report the synthesis and fabrication of Ti_2_O-based SAs and present the first systematic investigation of their performance in broadband ultrafast fiber lasers. Specifically, phase-pure Ti_2_O crystals were synthesized via solid-state sintering. High-performance Ti_2_O SAs were then fabricated through a photodeposition method. The balanced synchronous twin-detector measurement method demonstrated that Ti_2_O exhibited obvious and stable saturable absorption behavior. To validate their broadband mode-locking capability, the as-prepared Ti_2_O SAs were integrated into the Yb-doped and Er-doped fiber lasers, respectively. Experimental results show that both laser systems deliver stable pulsed output, with pulse durations of 441.7 ps at 1 μm and 522.5 fs at 1.5 μm. This work pioneers the application of Ti_2_O in ultrafast photonics, and provides an important reference and novel research insights for the design and development of advanced broadband optical devices and systems.

## 1. Introduction

Renowned for their ultrashort pulse widths, high peak power, compact design, and outstanding stability, ultrafast fiber lasers play a pivotal role in applications spanning material processing, optical communications, and biomedicine [1,2,3,4,5]. Passive mode-locking is the primary method used to produce ultrafast pulses in fiber lasers, where saturable absorbers (SAs) act as the core components to enable the formation and stabilization of ultrashort pulses [6,7]. An ideal SA should possess broadband response, high modulation depth, low saturation intensity, minimal non-saturable loss, and robust environmental stability [8,9]. Among these, modulation depth directly governs the pulse shortening capability, while saturation intensity determines the self-starting threshold and stability against environmental perturbations. In recent years, a diverse range of materials have been explored as SAs, including semiconductor saturable absorber mirrors (SESAMs) [10], graphene [11], black phosphorus [12], MXenes [13], bismuthene [14], etc. However, these SAs have many limitations: the narrow operating bandwidth and the complex fabrication of SESAMs, the relatively low modulation depth of graphene, and the rapid ambient degradation of black phosphorus. These limitations motivate the need for further exploration of novel SAs.

In the search for novel SAs with balanced performance, transition metal oxides (TMOs) have recently emerged as promising candidates due to their diverse electronic structures, tunable bandgaps, and strong light–matter interactions [15,16]. Ahmed et al. systematically investigated the application of titanium dioxide (TiO_2_) as a SA in fiber lasers at 2 μm [17], and Zhao et al. utilized α-Fe_2_O_3_ as a SA in EDFL to achieve both conventional soliton mode-locking and dissipative soliton resonance mode-locking [18]. In addition, other TMOs such as WO_3_ [19,20], NiO [21,22], CuO [23], Cr_2_O_3_ [24] have also been employed for mode-locking by groups from different countries. However, among Ti-based materials, the wide bandgap of TiO_2_ (~3.2 eV) restricts its intrinsic optical absorption almost exclusively to the ultraviolet region [25]. To address this limitation, a new class of sub-stoichiometric titanium oxides, specifically the Ti_2_O, has recently attracted considerable theoretical interest. In 2022, Yan et al. employed first-principles calculations to reveal that Ti_2_O and its halogen-functionalized derivatives (e.g., Ti_2_OF_2_, Ti_2_OCl_2_, Ti_2_OFCl) exhibit broadband optical absorption spanning from the ultraviolet to the near-infrared region [26,27,28,29,30]. These materials also feature tunable direct bandgaps in the range of 0.58–1.18 eV [29] with prolonged carrier lifetimes up to 2.8 ns [31]. Such properties are desirable for Sas; for instance, broadband absorption and a tunable direct bandgap ensure that the material maintains high optical absorption and modulation capability while operating at multiple wavelengths [32]. A longer carrier lifetime means that the photoexcited electron-hole pairs exist for a longer time before recombination, which makes it easier to fill the energy levels (i.e., achieve absorption saturation) at a lower optical fluence, thereby significantly reducing the saturation fluence [33]. While these theoretical predictions are promising, density functional theory (DFT) calculations at the generalized gradient approximation (GGA) level are known to underestimate bandgaps, and the influence of intrinsic defects on nonlinear optical properties has not been theoretically addressed. Moreover, unlike the well-established wet-chemical synthesis of TiO_2_, reliable fabrication routes for phase-pure Ti_2_O remain underdeveloped. Currently, only Fan et al. utilized pulsed laser deposition (PLD) technology to successfully fabricate hexagonal Ti_2_O on α-Al_2_O_3_ (0001) substrates under low oxygen pressures using a pure Ti target in 2019 [34]. Compared with the PLD method, which is substrate-dependent and yields thin films that are difficult to integrate directly into fiber systems, our solid-state sintering route produces bulk Ti_2_O crystals that can be readily ground into powder and deposited onto tapered fibers via a simple photodeposition process, offering better scalability and compatibility with fiber laser cavities. Prior to this study, no experimental validation in mode-locked fiber lasers had been reported. The gap between theoretical promise and experimental validation motivates the present work, in which we aim to systematically investigate the nonlinear optical properties of bulk Ti_2_O and demonstrate its mode-locking capability in fiber laser systems.

In this work, multiple characterization techniques were employed to confirm the successful synthesis of Ti_2_O crystals. Moreover, density functional theory (DFT) calculations were performed to analyze band structure and intrinsic optical properties. The balanced synchronous twin-detector measurement method confirmed that Ti_2_O exhibits an excellent nonlinear optical response, with modulation depths of 52.4% at 1 μm and 16.4% at 1.5 μm. These modulation depths are comparable to or better than many reported nonlinear optical materials, indicating its strong suitability for passive mode-locking applications. Based on these results, we hypothesize that Ti_2_O will exhibit broadband saturable absorption at both the 1 μm and 1.5 μm bands, and can be applied as a high-performance SA to achieve stable passive mode-locking in both Yb-doped fiber lasers (YDFL) and Er-doped fiber lasers (EDFL) systems. Therefore, we integrated Ti_2_O-based SAs into YDFL and EDFL for the first time, achieving stable mode-locked pulse outputs at 1 μm and 1.5 μm, respectively. This study fills the experimental gap in the optical properties of Ti_2_O. Furthermore, it establishes a solid foundation for the development of high-performance photonic devices based on titanium oxide materials.

## 2. Experimental Section

### 2.1. Fabrication of Ti_2_O

Ti_2_O crystals were prepared by a solid-state reaction employing elemental Ti and TiO_2_ powders as precursors. High-purity Ti powder (purity > 99%) and TiO_2_ powder (purity > 99%) were weighed according to a Ti/O atomic ratio of 2:1 and thoroughly mixed. The resulting mixture was uniaxially pressed into tablets under a pressure of 300 MPa, followed by calcination in a tube furnace at 1250 °C for 2 h under a flowing high-purity argon atmosphere, with a heating rate of 5 °C/min. After naturally cooling to room temperature, the tablet was polished with a sandpaper to remove the impurities at the surface, and the product was ground using an agate mortar to obtain Ti_2_O powder.

### 2.2. Characterization

The surface morphology of the as-prepared Ti_2_O samples was characterized using an electron microscope (Phenom Pharos G2, Thermo Fisher Scientific, Waltham, MA, USA). Transmission electron microscopy (TEM, JEM-F200, JEOL Ltd., Akishima, Tokyo, Japan) was further utilized to examine the microstructural details of the Ti_2_O. Energy-dispersive X-ray spectroscopy (EDS, JED-2300T, JEOL Ltd., Akishima, Tokyo, Japan) was used to measure the energy spectrum. X-ray diffraction (XRD) measurement was conducted using the Bruker D8 Advance instrument (Bruker AXS SE, Karlsruhe, Germany). The X-ray photoelectron spectroscopy (XPS) data was obtained through measurement using the relevant equipment (Thermo Fisher XPS K-Alpha, Waltham, MA, USA). The Raman spectra were collected with a Raman spectrometer (Renishaw plc, Wotton-under-Edge, Gloucestershire, UK). The linear optical absorption properties were recorded at room temperature with a UV-Vis-NIR spectrometer (UV-3600 Plus, Shimadzu Corporation, Kyoto, Japan).

### 2.3. Computational Methods

The calculations were conducted using the Vienna Ab initio Simulation Package (VASP 6.1) with the projector-augmented wave (PAW) method [35,36]. The exchange-correlation potentials were considered on the basis of generalized gradient approximation, formalized by Perdew–Burke–Ernzerhof [37]. A plane-wave cutoff energy of 550 eV and a Γ-centered k-point grid of 21 × 21 × 13 were employed in the calculations. The optB88-vdw method was used to model the van der Waals interaction [38]. The crystal structure was fully relaxed until the total energy and atomic forces were smaller than 10^-5^ eV and 0.01 eV/Å, respectively. The linear optical properties were obtained from the frequency-dependent dielectric function *ε*(*ω*)

(1) $$\varepsilon (\omega )=\varepsilon_{1}(\omega )+i\varepsilon_{2}(\omega )$$

where *ε*_1_(*ω*) and *ε*_2_(*ω*) are the real and imaginary parts of the dielectric function, and *ω* is the photon frequency. In the framework of the one-electron picture, the imaginary part of the dielectric function *ε*_2_(*ω*) is obtained by:

(2) $$\begin{array}{cc} \varepsilon_{2}(\omega )= & \frac{4\pi^{2}e^{2}}{\Omega }\underset{q\to 0}{lim}\;\frac{1}{q^{2}}\\ & \times \sum_{c,\nu ,k}\;2w_{k}\delta \left(E_{c}-E_{\nu }-\omega \right)|\langle c|e\cdot q|\nu \rangle {|}^{2} \end{array}$$

where *<c|**e**·**q**|v>* denotes the integrated optical transitions from the valence states (*v*) to the conduction states (*c*), *e* is the polarization direction of the photon and *q* is the electron momentum operator. The integration over *k* is performed by summation over special *k* points with a corresponding weighting factor *w_**k**_*. In addition, the real part of the dielectric function *ε*_1_(*ω*) can be determined from the Kramers–Kronig relation given by [39]:

(3) $$\varepsilon_{1}(\omega )=1+\frac{2}{\pi }P\int_{0}^{\infty }\;\frac{\varepsilon_{2}\left(\omega^{\prime }\right)\omega^{\prime }}{\omega^{\prime 2}-\omega^{2}+i\eta }d\omega^{\prime }$$

where *P* indicates the principal value and *η* is the complex shift parameter. Therefore, the frequency-dependent linear optical spectra of absorption coefficient *α*(*ω*) can be simulated from the real *ε*_1_(*ω*) and the imaginary *ε*_2_(*ω*) parts [40]:

(4) $$\alpha \left(\omega \right)=\frac{\sqrt{2}\omega }{c}{\left(\sqrt{\varepsilon_{1}^{2}+\varepsilon_{2}^{2}}-\varepsilon_{1}\right)}^{\frac{1}{2}}$$

### 2.4. Fabrication of Ti_2_O SA

Ti_2_O-based SAs on tapered optical fibers were fabricated via a photodeposition method. First, a small amount of Ti_2_O powder was dispersed in 10 mL of isopropanol solution, followed by ultrasonic treatment at 40 kHz for 10 h. After ultrasonic treatment, the above dispersion was allowed to stand for 1 h to obtain a uniformly dispersed Ti_2_O suspension. Second, a conventional single-mode fiber was tapered via a hydrogen–oxygen flame melting and stretching method, yielding a tapered fiber with a length of 26 mm and an insertion loss of 0.1 dB. Finally, photodeposition method was employed to adsorb Ti_2_O onto the surface of the tapered fiber. During the preparation process, the Ti_2_O material in the tapered region interacts strongly with the evanescent field, thereby adsorbing onto the optical fiber to form a Ti_2_O SA. By monitoring the optical power at the output end of the tapered fiber in real time, the output power was tuned to 83 mW (for the 1 μm) and 44 mW (for the 1.5 μm) to fabricate Ti_2_O SAs suitable for fiber lasers operating at 1 μm and 1.5 μm bands.

### 2.5. Fiber Laser

The ring cavity configuration consisted of a 980 nm pump laser, a wavelength division multiplexer (WDM), a polarization-insensitive optical isolator (ISO), and a polarization controller (PC). An 80/20 optical coupler (OC) and 0.75 m of YDF gain fiber were used for the YDFL. A 90/10 OC and 0.5 m EDF gain fiber were used for the EDFL. During the test, pulse trains were observed via an oscilloscope (EXR104A, Keysight Technologies, Inc., Santa Rosa, CA, USA), and optical spectra were captured via a spectrometer (AQ6370D, Yokogawa Electric Corporation, Tokyo, Japan). For the EDFL system, pulse duration was measured using an autocorrelator (APE-150 PulseCheck, APE GmbH, Berlin, Germany). For the YDFL system, since the output pulse duration exceeded the maximum measurement range of the autocorrelator, an oscilloscope (EXR104A, Keysight Technologies, Inc., Santa Rosa, CA, USA) was used to directly record the envelope of single pulses. The RF spectrum was measured using a spectrum analyzer (N9332C, Keysight Technologies, Inc., Santa Rosa, CA, USA).

## 3. Results and Discussion

**Figure 1:**
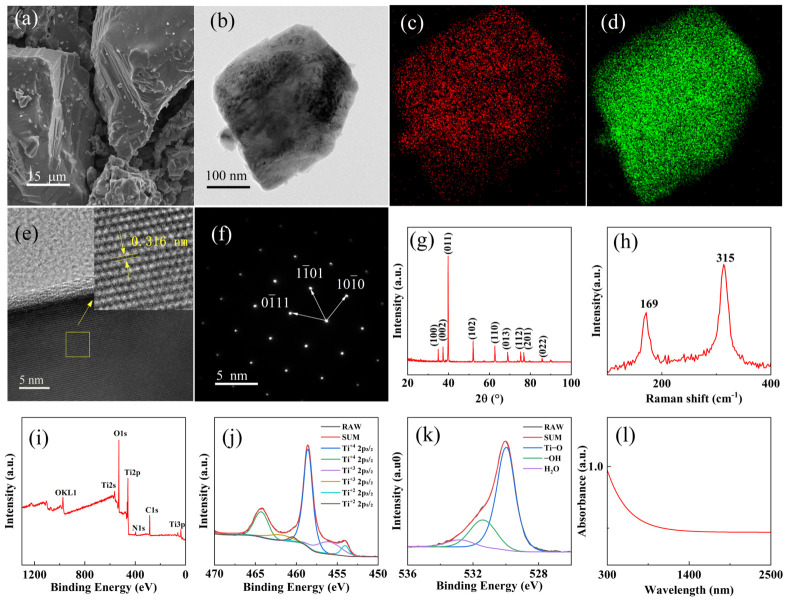
Morphology and structural characterization of Ti_2_O. (**a**) SEM image. (**b**) TEM image. (**c**,**d**) EDS. (**e**) HRTEM image. (**f**) SAED. (**g**) XRD pattern. (**h**) Raman spectrum. (**i**) Full-scan XPS spectra. (**j**) High-resolution scans of Ti(2p). (**k**) High-resolution scans of O(1s). (**l**) Linear optical absorption spectrum.

Figure 1a is the scanning electron microscope (SEM) image. The synthesized Ti_2_O exhibits a micrometer-scale layered structure with a lateral size of approximately 15 μm. Figure 1b shows a TEM bright field image, demonstrating excellent dispersion of the samples, with no apparent agglomeration observed. As shown in Figure 1c,d, the EDS mapping shows uniform distribution of Ti and O at the micrometer scale, ruling out obvious phase separation and confirming the homogeneous formation of the Ti_2_O phase. As presented in Figure 1e, high-resolution TEM (HRTEM) confirms the high crystallinity of Ti_2_O nanoparticles, with distinct lattice fringes of 0.316 nm interplanar spacing corresponding to the (110) crystal plane of Ti_2_O. Selected area electron diffraction (SAED, crystal zone axis [$01\bar{1}1$]) patterns further verify the crystalline nature of Ti_2_O, as shown in Figure 1f. The XRD measurements verify the crystal phase and purity of Ti_2_O, as shown in Figure 1g. All diffraction peaks can be indexed to the Ti_2_O structure [(111), (200), (220), (311), (222)] (PDF#01-073-1116), with no characteristic peaks of impurity phases (e.g., TiO_2_, Ti_3_O_5_) detected. As shown in Figure 1h, the Raman spectroscopy confirms the formation of Ti_2_O, with characteristic peaks at 169 cm^−1^ (F2g mode) and 315 cm^−1^ (A1g mode). The XPS analysis was performed to investigate the surface chemical states and composition of Ti_2_O. The full-scan XPS spectra (Figure 1i) confirm the presence of Ti and O, along with a small amount of adventitious carbon. Deconvolution of the high-resolution Ti(2p) spectrum (Figure 1j) reveals three sets of doublets corresponding to Ti^4+^, Ti^3+^, and Ti^2+^ species [41]. The dominant doublet at 459.0/464.8 eV is assigned to Ti^4+^, while the weaker doublets at 456.5/462.3 eV and 455.2/461.0 eV correspond to Ti^3+^ and Ti^2+^, respectively. The presence of Ti^2+^ and Ti^3+^ alongside the dominant Ti^4+^ signal confirms a mixed-valence state, which is commonly induced by oxygen vacancies in sub-stoichiometric titanium oxides. It has been theoretically and experimentally demonstrated that oxygen vacancies and the resulting mixed-valence Ti ions (Ti^2+^/Ti^3+^) introduce a continuum of intra-gap localized states in titanium oxide systems [42]. For the Ti_2_O SA in this work, the coexistence of Ti^4+^, Ti^3+^, and Ti^2+^ (Figure 1j) indicates a high density of such defect states. These localized states can act as independent saturable absorption centers and provide additional low-energy optical transition channels, enhancing the nonlinear optical response in the near-infrared region. Moreover, the prolonged excited-state lifetimes associated with defect-trapped carriers contribute to the reduced saturation intensity, which is consistent with the low saturation powers measured in our experiments. Figure 1k is the O(1s) spectrum, which was fitted with lattice oxygen (530.2 eV), surface hydroxyl groups (531.6 eV), and adsorbed water molecules (533.1 eV). The presence of adsorbed water is due to the sample surface being briefly exposed to the ambient environment prior to measurement. The optical properties of Ti_2_O were evaluated via an ultraviolet–visible–infrared (UV-Vis-NIR) spectrometer, as shown in Figure 1l. Broadband optical absorption has been observed across the visible to near-infrared spectrum, making Ti_2_O a promising candidate for broadband photonic and photoelectronic applications.

**Figure 2:**
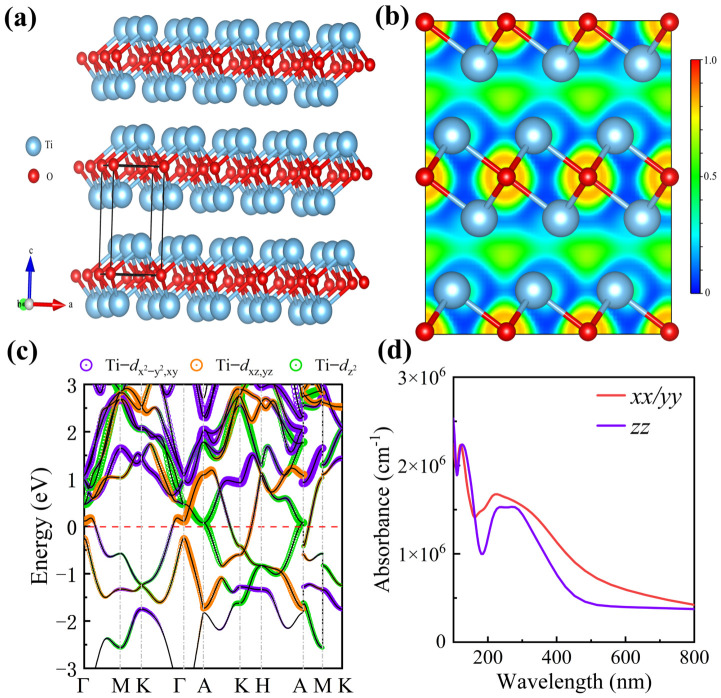
Theoretical calculation of Ti_2_O. (**a**) Crystal of bulk Ti_2_O. The black line indicates the primitive cell. (**b**) ELF of Ti_2_O. (**c**) Pband of Ti_2_O. (**d**) Optical absorption spectrum of Ti_2_O.

The optimized crystal structure of bulk Ti_2_O is illustrated in Figure 2a, revealing a hexagonal lattice that aligns well with the XRD results discussed earlier. The optimized lattice constants of bulk Ti_2_O are a = b = 2.97 Å, c = 4.82 Å, which are in accordance with experimental values of bulk Ti_2_O (a = 2.96 Å, c = 4.83 Å) [43]. To analyze the nature of the chemical bonding and interatomic interactions, we calculated the electron localization function (ELF) [44], as depicted in Figure 2b. The ELF map provides a clear visualization of electron distribution. Here, the values of 1.0 and 0.5 represent fully localized and fully delocalized electrons, respectively. The distinct electron localization observed between Ti and O atoms confirms a significant ionic bonding character within the layers. Crucially, the ELF map shows an absence of electron localization between the individual layers, thereby confirming the van der Waals nature of the interlayer bonding. This weak interlayer coupling is beneficial for material exfoliation and for ensuring stable interaction with the evanescent field of the tapered fiber.

The electronic band structures of Ti_2_O were further investigated, and the projected band structure (Pband) is presented in Figure 2c. The Pband reveals that Ti_2_O exhibits a metallic characteristic with several bands crossing the Fermi level. This band structure favors broadband light absorption, as it allows for electron transitions across a wide range of energies from the visible to the near-infrared region. The steep band dispersion near the Fermi level suggests a small effective mass of charge carriers, which facilitates fast carrier relaxation, a critical property for ultrafast SAs.

Finally, to corroborate the experimental linear optical absorption spectrum (Figure 1l), we calculated the frequency-dependent dielectric function and derived the optical absorption spectrum of Ti_2_O, shown in Figure 2d. The simulated spectrum demonstrates a robust and broad absorption band spanning from the ultraviolet to the near-infrared region, with a prominent absorption tail extending well beyond 800 nm. This broadband absorption characteristic matches our UV-Vis-NIR experimental results. The calculated and measured optical responses show good consistency. This suggests that the saturable absorption we observed is indeed due to the intrinsic electronic structure of Ti_2_O. The presence of in-gap defect states (as suggested by XPS and Raman) combined with the intrinsic band structure calculated here synergistically contributes to the broad and efficient nonlinear optical response, positioning Ti_2_O as an exceptional candidate for broadband photonic applications.

This metallic band structure enables optical transitions across a wide energy range near the Fermi level, giving rise to the Pauli blocking effect that serves as a well-established mechanism for saturable absorption in metallic and semi-metallic systems [11]. The in-gap defect states induced by oxygen vacancies (as evidenced by the Ti^2+^/Ti^3+^ species in XPS) are also expected to contribute additional saturable absorption pathways [42].

**Figure 3:**
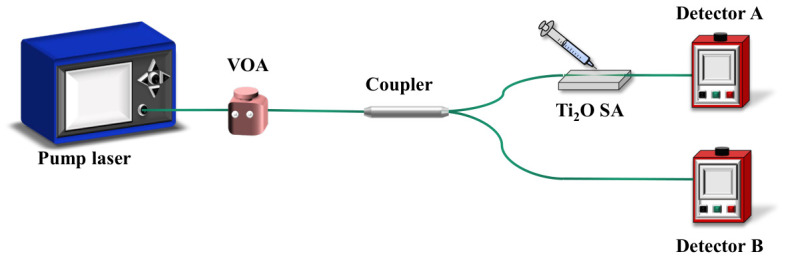
The schematic diagram of the balanced synchronous twin-detector measurement system.

**Figure 4:**
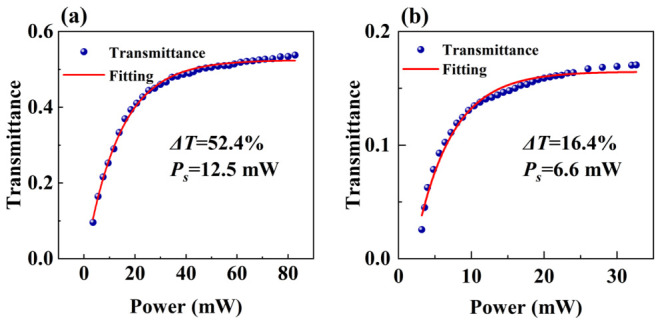
Nonlinear transmission response of Ti_2_O at 1 µm (**a**) and 1.5 µm (**b**).

The nonlinear absorption response of the as-prepared Ti_2_O SA was systematically investigated via the balanced synchronous twin-detector measurement technique. Figure 3 shows the experimental setup. A YDFL served as the 1 μm band source, and an EDFL provided the 1.5 μm band. The laser power was regulated via a variable optical attenuator (VOA), and the laser beam was split by a 50:50 OC. One beam was directed to an optical power meter as a reference, and the other was transmitted through the Ti_2_O SA and detected by the second power meter. The transmittance of the Ti_2_O SAs was calculated as the ratio of the two power meter readings. Figure 4a,b show the results for the 1 μm and 1.5 μm bands, respectively. The transmittance of the Ti_2_O SAs increases gradually and eventually saturates with increasing incident laser intensity, exhibiting typical saturable absorption behavior.

The dependence of transmittance (*T*) on incident power (*P*) was described by the standard saturable absorption expression:

(5) $$T=1-\Delta T^{*}exp\left(-P/P_{s}\right)-\alpha_{ns}$$

where *ΔT* is the modulation depth, *Ps* is the saturation power, and *α*_*ns*_ is the non-saturable loss. Fitting results show that the Ti_2_O SA has modulation depths of 52.4% (1 μm) and 16.4% (1.5 μm), with saturation powers of 12.5 mW (1 μm) and 6.6 mW (1.5 μm), respectively. Table 1 presents a comparison of the modulation depth between Ti_2_O and other materials under the same measurement method. It should be noted that the modulation depth values quoted here are extracted from different studies using various measurement conditions (e.g., pulse width, wavelength, intensity), and therefore a direct quantitative comparison should be interpreted with caution. Nonetheless, the high values observed for Ti_2_O under our standardized measurement conditions clearly indicate its competitive saturable absorption capability.

**Figure 5:**
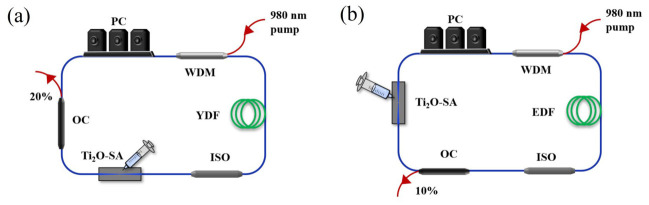
Schematic of the Ti_2_O-based mode-locking (**a**) YDFL;(**b**) EDFL.

Given the broadband absorption and saturable absorption properties of Ti_2_O, we integrated the as-prepared Ti_2_O SAs into 1 μm YDFL and 1.5 μm EDFL to explore their ultrafast photonics applications. Figure 5 presents a schematic of the YDFL and EDFL. The Ti_2_O SAs were integrated into both ring cavities to construct complete passive mode-locked laser systems. To obtain a stable mode-locked output, the intracavity polarization state was adjusted by rotating the PC and the pump power was synergistically optimized in both systems, and ultimately stable laser pulse trains and spectral output were obtained.

**Figure 6:**
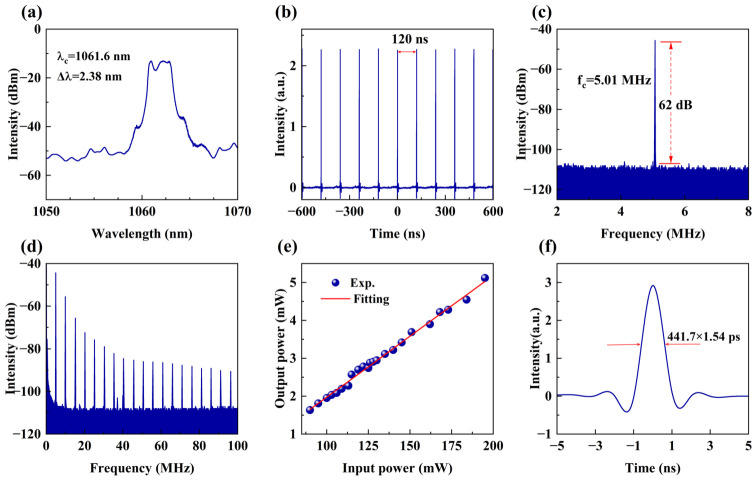
Output characteristics of YDFL with integrated Ti_2_O SA. (**a**) Optical output spectrum. (**b**) Corresponding pulse train. (**c**) Fundamental RF spectrum. (**d**) RF spectrum measured over a 100 MHz span. (**e**) Output power versus pump power. (**f**) Individual pulse profile.

The mode-locking performance of the 1 μm YDFL is summarized in Figure 6. The relevant data were recorded at a pump power of 100 mW. In Figure 6a, the laser delivers output pulses with a central wavelength of 1061.6 nm and a 3 dB spectral bandwidth of 2.38 nm. Figure 6b shows the corresponding pulse train, with a pulse interval of 120 ns. As shown in Figure 6c, RF spectrum analysis reveals that the laser has a central frequency of 5.01 MHz, in good agreement with the physical cavity length (42 m), and the signal-to-noise ratio (SNR) of the output pulses is as high as 62 dB. Further spectral noise analysis, shown in Figure 6d, reveals no obvious spurious signals in the 0–100 MHz range, fully confirming stable passive mode-locking operation of the Ti_2_O SA-integrated YDFL system. As shown in Figure 6e, stable mode-locked operation was achieved with a pump power ranging from 90 mW to 195 mW. At the maximum pump power, the output power was 5.1 mW, yielding a single-pulse energy of 1.03 nJ. Figure 6f shows a single-pulse profile with a pulse width of 441.7 ps. The time-bandwidth product (TBP) was calculated to be 280. The relatively high TBP indicates significant chirp in the output mode-locked pulses, which is consistent with the characteristics of all-normal dispersion YDFL cavities [53].

**Figure 7:**
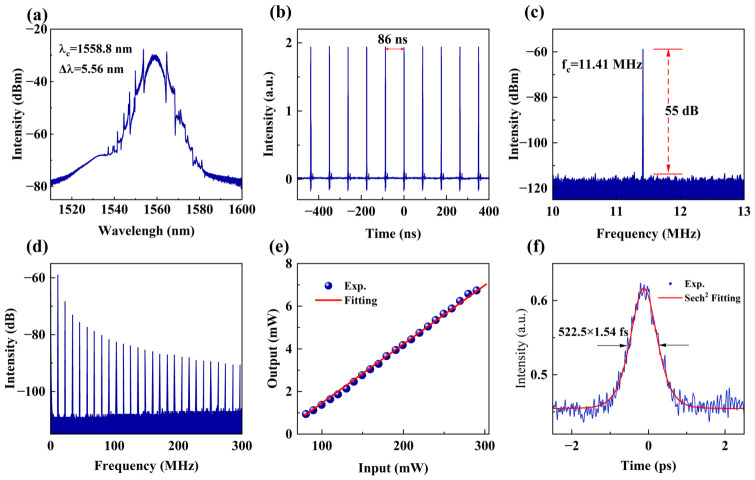
Output characteristics of EDFL with integrated Ti_2_O SA. (**a**) Optical output spectrum. (**b**) Corresponding pulse train. (**c**) Fundamental RF spectrum. (**d**) RF spectrum measured over a 300 MHz span. (**e**) Output power versus pump power. (**f**) Autocorrelation trace with a Sech2 fitting.

The mode-locked output characteristics of the 1.5 μm EDFL are shown in Figure 7, where the relevant data were recorded at a pump power of 90 mW. In Figure 7a, obvious Kelly sidebands are clearly observed in the output spectrum, a typical characteristic of soliton mode-locking [54,55], confirming that the EDFL system operates stably in the soliton mode-locking state. The output pulse has a central wavelength of 1558.8 nm and a 3 dB spectral bandwidth of 5.56 nm. Figure 7b shows the corresponding pulse train, with a pulse interval of 86 ns. As shown in Figure 7c, the central frequency of the pulse train is 11.41 MHz, which is completely consistent with the theoretically calculated value of the designed ring resonant cavity length (18 m), and the SNR of the output pulse reaches 55 dB. In Figure 7d, no obvious spurious signals are detected in the frequency range of 0–300 MHz, further verifying the stability of the mode-locking operation of the system. As presented in Figure 7e, the minimum pump power required to achieve a stable mode-locked pulse train was measured to be 85 mW. The pump power could be increased up to a maximum of 290 mW, at which the output power was 6.9 mW, corresponding to a single-pulse energy of 0.61 nJ. In Figure 7f, the pulse duration is 522.5 fs obtained by fitting the pulse envelope with a Sech2 function, with a corresponding TBP of 0.358. This TBP (0.358) is close to the Sech2 transform limit (0.315), suggesting that the pulses are nearly chirp-free and of high quality.

Furthermore, to comprehensively evaluate the performance of the Ti_2_O SA, we compared the YDFL and EDFL based on Ti_2_O with those based on previously reported typical SAs (Table 2). Comparative analysis shows that the Ti_2_O SA enables high-performance short-pulse outputs in both YDFL and EDFL systems, featuring high pulse energy and relatively short pulse duration. As an SA material, it exhibits great potential for application and development in the field of ultrafast fiber lasers.

## 4. Conclusions

In this study, Ti_2_O was successfully synthesized via a solid-state sintering method, and its structural and optical properties were systematically characterized. Combined with theoretical calculations, the mode-locking capability of Ti_2_O in EDFL and YDFL was demonstrated. Experimental results show that stable passively mode-locked pulse outputs were achieved at the 1 μm and 1.5 μm bands using Ti_2_O-based SAs, delivering ultrashort pulses of 441.7 ps and 522.5 fs, respectively. This work is the first to apply Ti_2_O to ultrafast laser systems, confirming its potential as a broadband nonlinear optical material. Given the significant prospects of ultrafast lasers in optical communications, precision manufacturing, and biomedicine, the further development of Ti_2_O-based photonic devices is expected to advance related applications, and their optical properties and modulation mechanisms warrant further in-depth exploration in future studies.

## Figures and Tables

**Table 1:** Ti_2_O SA versus other SAs at 1 µm and 1.5 µm measured under the balanced synchronous twin-detector measurement.

Wavelength	SA	Modulation Depth	Ref.
1 μm	Graphene	8%	[45]
	BP	12.5%	[46]
	Nb_4_C_3_	22%	[47]
	Ti_2_CBr_2_	48.25%	[13]
	FeOCl	2.81%	[48]
	Ti_3_C_2_T_X_/MoO_3_	13.7%	[49]
	Ti_2_O	52.4%	This work
1.5 μm	Graphene	1.3%	[11]
	Bi	2.2%	[50]
	ZrS_2_	6.3%	[51]
	Ti_3_C_2_	7.19%	[52]
	WO_3_	1.85%	[19]
	FeOCl	2.67%	[48]
	CuO	1.97%	[23]
	α-Fe_2_O_3_	4.2%	[18]
	Ti_2_O	16.4%	This work

**Table 2:** Comparison of mode-locking performances for various SAs in YDFL and EDFL.

	Materials	Center Wavelength (nm)	Repetition Rate (MHz)	Pulse Duration (ps)	Pulse Energy (nJ)	Ref.
YDFL	BP	1064.4	16.77	51	1.13	[46]
	Graphene	1069.8	0.9	580	0.41	[45]
	MoS_2_	1034.1	6.74	656	0.35	[56]
	Nb_2_C	1031.5	14.8	271	0.89	[57]
	Ti_3_C_2_Tx	1065.8	18.96	480	0.47	[58]
	Ti_2_CT_x_	1037.8	16.5	792	0.72	[59]
	FeOCl	1034.1	8.709	0.572	0.068	[48]
	Ti_3_C_2_T_X_/MoO_3_	1031	24.2	12.7	4.1	[49]
	CuO	1038.5	7.99	1260	-	[23]
	Ti_2_O	1061.6	5.01	441.7	1.03	This work
EDFL	BP	1576.1	34.27	0.4037	0.055	[46]
	Graphene	1559.1	25.67	0.433	0.05	[60]
	MoS_2_	1552	12.99	0.960	0.065	[61]
	Nb_2_C	1559.98	12.54	0.603	0.78	[62]
	Ti_2_CT_x_	1530.85	8.46	0.265	1.44	[59]
	FeOCl	1556.8	5.411	1.11	0.81	[48]
	ZrO_2_/SiO_2_	1562.8	6.55	0.964	2.25	[63]
	WO_3_	1556.22	9.52	0.961	4.0015	[19]
	NiO	1561.27	8.0	0.793	0.89	[22]
	α-Fe_2_O_3_	1560.3	6.53	1.13	12	[18]
	CuO	1552	7.32	1.3	1.12	[23]
	Ti_2_O	1558.8	11.41	0.5225	0.61	This work

## Data Availability

The original contributions presented in this study are included in the article. Further inquiries can be directed to the corresponding authors.
